# Draft Genome Sequence of *Thermoanaerobacterium saccharolyticum* Strain NTOU1, a Thermophilic Bacterium Isolated from Marine Shallow Hydrothermal Vents

**DOI:** 10.1128/genomeA.01019-14

**Published:** 2014-10-09

**Authors:** Engkong Tan, Yusan Chen, Jung Kuan, Chiajui Lin, S. S. S. De S. Jagoda, Fupang Lin, Wenshyong Tzou, Shigeharu Kinoshita, Shugo Watabe, Shuichi Asakawa, Shiumei Liu

**Affiliations:** a Laboratory of Aquatic Molecular Biology and Biotechnology, Department of Aquatic Bioscience, Graduate School of Agricultural and Life Sciences, the University of Tokyo, Tokyo, Japan; b Institute of Bioscience and Biotechnology, National Taiwan Ocean University, Keelung, Taiwan; c School of Marine Biosciences, Kitasato University, Kanagawa, Japan

## Abstract

*Thermoanaerobacterium saccharolyticum* strain NTOU1 has the ability to utilize several kinds of sugars in lignocellulosic biomass to produce ethanol more efficiently than other bacteria. Here, we report the draft genome sequence and annotation of this strain, which may provide insights into the possible genes and metabolic pathways related to ethanol production.

## GENOME ANNOUNCEMENT

As an alternative to fossil fuels, the biofuel-like ethanol produced from bacteria has received great interest (1). Converting biomass to sugars is required but is an expensive step in biofuel production (2). One way to solve this problem is to use thermophilic bacteria to produce ethanol (3), as it works with a shorter fermentation time at higher temperatures. Bacteria belonging to the genus *Thermoanaerobacterium* have the ability to ferment both pentose and hexose components in hemicelluloses, and ethanol is generated as a by-product during fermentation at 70°C (4). *Thermoanaerobacterium saccharolyticum* strain NTOU1, a Gram-negative thermophilic bacterium, was isolated from shallow marine hydrothermal vents off Gueishandao Island in Taiwan. Strain NTOU1 is of particular interest, because it has been reported that among wild-type anaerobic thermophilic ethanogens, this strain had the highest ethanol yield rate (5). Here, we attempted to sequence, assemble, and annotate the draft genome sequence of NTOU1.

We performed whole-genome shotgun sequencing using the Ion Torrent PGM sequencer (Life Technologies). To achieve the best sequencing result, a 400-bp sequencing kit and 318 Chip (Life Technologies, Japan) were selected. *De novo* genome assembly was done by MIRA software version 3.9.18 (http://sourceforge.net/projects/mira-assembler/), and the assembled genome sequence was then annotated with Rapid Annotations using Subsystems Technology (RAST) 4.0 (6). Furthermore, rRNAs and tRNAs were predicted by RNAmmer 1.2 (7) and tRNAscan-SE 1.21 (8), respectively.

A total of 826,957,387 bp (2,694,361 reads) were sequenced and applied to MIRA. One hundred one contigs of >500 bp were assembled, and the total length is 2,833,212 bp, with an *N*_50_ of 59,076 bp. The average G+C content is 34.84%. The NTOU1 draft genome sequence showed approximately similar total lengths and G+C contents as the available genomes of the same species (*T. saccharolyticum* JW/SL-YS485, GenBank accession no. NC_017992; genome size, 2.88 Mb, G+C content, 35.1%). Annotation revealed that NTOU1 contains 3,101 protein-coding genes, 7 rRNAs, and 54 tRNAs. Furthermore, 21 protein-coding sequences related to xylose utilization, including xylose isomerase, xylulose kinase, and beta-xylosidase, 20 coding sequences related to mannose metabolism and 13 coding sequences related to l-arabinose utilization were also annotated by RAST. This draft genome may provide insight into the possible genes and metabolic pathways related to ethanol production. Also, a Pacific Biosciences RSII sequencing and assembly of this strain is ongoing, with the hope of achieving the complete genomic sequence of this strain.

### Nucleotide sequence accession numbers.

The accession numbers of this draft genome sequence in DDBJ/EMBL/GenBank are BBKT01000001 to BBKT01000101.
